# From Local Adaptation to Ecological Speciation in Copepod Populations from Neighboring Lakes

**DOI:** 10.1371/journal.pone.0125524

**Published:** 2015-04-27

**Authors:** Omar Alfredo Barrera-Moreno, Jorge Ciros-Pérez, Elizabeth Ortega-Mayagoitia, José Arturo Alcántara-Rodríguez, Elías Piedra-Ibarra

**Affiliations:** 1 Programa de Doctorado en Ciencias del Mar y Limnología, Facultad de Estudios Superiores Iztacala, Universidad Nacional Autónoma de México, Tlalnepantla, Estado de México, México; 2 Proyecto de Investigación en Limnología Tropical, Facultad de Estudios Superiores Iztacala, Universidad Nacional Autónoma de México, Tlalnepantla, Estado de México, México; 3 Programa de Doctorado en Ciencias Biológicas, Facultad de Estudios Superiores Iztacala, Universidad Nacional Autónoma de México, Tlalnepantla, Estado de México, México; 4 Laboratorio de Fisiología Vegetal, UBIPRO, Facultad de Estudios Superiores Iztacala, Universidad Nacional Autónoma de México, Tlalnepantla, Estado de México, México; University of Arkansas, UNITED STATES

## Abstract

Continental copepods have been derived from several independent invasive events from the sea, but the subsequent evolutionary processes that account for the current diversity in lacustrine environments are virtually unknown. Salinity is highly variable among lakes and constitutes a source of divergent selection driving potential reproductive isolation. We studied four populations of the calanoid copepod *Leptodiaptomus* cf. *sicilis* inhabiting four neighboring lakes with a common history (since the Late Pleistocene) located in the Oriental Basin, Mexico; one lake is shallow and varies in salinity periodically (1.4–10 g L^-1^), while three are deep and permanent, with constant salinity (0.5, 1.1 and 6.5 g L^-1^, respectively). We hypothesized that (1) these populations belong to a different species than *L*. *sicilis* sensu stricto and (2) are experiencing ecologically based divergence due to salinity differences. We assessed morphological and molecular (mtDNA) COI variation, as well as fitness differences and tests of reproductive isolation. Although relationships of the Mexican populations with *L*. *sicilis* s.s. could not be elucidated, we identified a clear pattern of divergent selection driven by salinity conditions. The four populations can still be considered a single biological species (sexual recognition and hybridization are still possible in laboratory conditions), but they have diverged into at least three different phenotypes: two locally adapted, specialized in the lakes of constant salinity (saline vs. freshwater), and an intermediate generalist phenotype inhabiting the temporary lake with fluctuating salinity. The specialized phenotypes are poorly suited as migrants, so prezygotic isolation due to immigrant inviability is highly probable. This implication was supported by molecular evidence that showed restricted gene flow, persistence of founder events, and a pattern of allopatric fragmentation. This study showed how ecologically based divergent selection may explain diversification patterns in lacustrine copepods.

## Introduction

Invasion of inland freshwater environments by marine species resulting in adaptive radiation is one of the “most dramatic evolutionary transitions in the history of life” [1]. A remarkable example of this is the invasion of copepods into continental waters. Continental lineages of copepods originated from 22 independent invasive waves from the sea that were followed by subsequent speciation events [2]. The result of these invasion-radiation events accounts for ~ 2800 freshwater species currently recognized. While the adaptation of marine copepods to brackish and freshwater conditions and genetic and physiological divergence among populations has been studied recently [1,3], study of evolutionary processes and patterns of diversification in lacustrine copepods remain scarce. Moreover, these investigations are usually linked to unraveling the cryptic diversity in this group and focus on genetic and morphological data [4–6]. Such evidence has been used to hypothesize that trophic specialization within a lake [7] and adaptation to different physicochemical and environmental settings among lakes [6] may promote divergence and speciation in pelagic copepods. Thus, divergence and reproductive isolation in this group may be a case of ecological speciation.

Lakes are environments where diversification rates are expected to be higher than in marine systems because each body of water is the result of a combination of unique factors including climatic regimes, regional geology, and tectonic activity. Thus, adaptation to different environments and the limited connectivity between lakes can result in frequent allopatric speciation [8]. Salinity is one of the most influential aquatic features, acting directly and indirectly on the growth, life history, distribution, and molecular evolution of aquatic organisms [9]. However, not all lakes possess salinities that qualify them as freshwater; the continuum of salinities ranges from < 0.1 to 350 g L^-1^ and beyond [10], constituting a potential source of divergent selection where ecological speciation can proceed [11].

Populations subjected to divergent natural selection can produce one or more specialized genotypes, adapted to alternative local conditions (disruptive selection) or generalist genotypes adapted to a wide range of conditions, exhibiting either uniform or plastic phenotypes [12]. When the effects of the environmental factor that promotes local adaptation are strong, immigrant individuals usually have a lower fitness compared to the local population and may be eliminated (i.e., immigrant inviability) thus reducing gene flow and thus constituting an effective reproductive barrier (premating isolation). This is the second step (of three) for complete ecological speciation [11]. Divergent adaptation to salinity could explain why many lacustrine species that were considered to be cosmopolitan with wide tolerances to salinity (generalists) are actually composed of a number of cryptic species adapted to narrower ranges and restricted distributions [9,13], even though generalist euryhaline species [14,15] truly exist. Moreover, some recent papers note the role of salinity as a source of divergent selection in continental aquatic habitats, promoting diversification and speciation in rotifers [16], gammarids [17], and fish [18–20].

We have studied a cluster of eight endorheic lakes located in the Oriental Basin (Cuenca Oriental) in central Mexico. This region is characterized by shallow, ephemeral, playa lakes (El Carmen, Tepeyahualco) and permanent/deep lakes (Quechulac, La Preciosa, Atexcac, Alchichica), ranging from freshwater to saline. Several microendemic species have been found including the following: a diatom, *Cyclotella alchichicana* [21], a rotifer, *Brachionus* sp. ‘Mexico’ [9], copepods *Leptodiaptomus garciai* [13] and *Cletocampus gomezi* [22], an amphipod, *Caecidotea williamsi* [23], an ostracod, *Limnocytherina axalapasco* [24], a salamander, *Ambystoma taylori*, and silverside fish, *Poblana*, including at least two species [25]. Local adaptation to salinity has been described for the rotifer *Brachionus* sp. ‘Mexico’ [9].

Four of these lakes (two deep, freshwater; one deep, saline; one shallow, ephemeral, and highly variable salinity) contain populations of a calanoid copepod that morphologically resembles *Leptodiaptomus sicilis* (Forbes 1886) [26,27]. This species has not been reported elsewhere in Mexico but seems to be widely distributed in the Laurentian Great Lakes and other regions within the United States of America and Canada, from freshwater up to 40 g L^-1^ [28]. It has been reported to be the dominant species in the zooplankton of both freshwater [29] and saline lakes (10–13 g L^-1^) [30,31]. However, there are several reasons that lead us to posit that *L*. *sicilis* may represent a complex of cryptic species, and that populations that inhabit Cuenca Oriental may belong to a different biological species from the populations described by Forbes. First, salinity differences promote high rates of divergence among aquatic organisms, as stated above. Second, given that the body plan of pelagic copepods is so successful and often dominates open waters [32], speciation events may produce minor morphological divergence between closely allied species [33]. Third, there is increasing evidence that the apparent distribution of copepod species across broad salinity ranges can be the outcome of the aggregated distribution of different genotypes or of distinct cryptic species [13,34]. Finally, more than 90% of freshwater copepod species are endemic to a single zoogeographic region [35]; in particular, the family Diaptomidae is characterized by species of very restricted distribution [33], and the diaptomid fauna of Mexico is no exception [36]. Moreover, given the environmental divergence among the four Mexican lakes in terms of salinity and habitat permanence, we also hypothesized that irrespective of their taxonomic identity, these populations are undergoing ecological speciation: We hypothesized that populations may have either locally adapted to deep permanent lakes or are generalist phenotypes inhabiting ephemeral playa lakes. If local adaptation has occurred, immigrants are expected to have lower fitness than local individuals and locally adapted genotypes also will be poorly adapted to foreign environments. Both circumstances will impair their capacity of effective dispersal and decrease the probability of gene flow, reinforcing the process of ecological speciation leading to reproductive incompatibility.

To test our hypotheses, we took an integrative approach to assess the degree of divergence among populations at several levels: phenotypic, genotypic and molecular clustering, lineage sorting and reproductive isolation [11]. Thus, we analyzed the following: (1) morphological divergence implicated in reproduction among *L*. *sicilis* from the Great Lakes and the Mexican populations; (2) sequence divergence in mitochondrial cytochrome *c* oxidase subunit I (COI) compared with other species to elucidate patterns of genetic structuring among Mexican populations; (3) life history differences including patterns of resting egg hatching, survivorship, development, and intrapopulation reproduction in reciprocal transplant experiments in order to reveal locally adapted/generalist genotypes and selection against immigrants (immigrant inviability); and (4) results of mating trials among three of the Mexican populations in a common garden experiment to evaluate the degree of reproductive compatibility.

## Materials and Methods

### Ethics statement

We collected copepods from four lakes in Mexico that are not under protection by Mexican laws. Further, Mexican zooplankton is not under protection laws as well; thus, no specific permissions were required to collect samples.

### Study area

The lakes studied are located in the Oriental Basin, Mexico, in the Transmexican Volcanic Belt, ≈ 2,300 m above sea level [37]. While they have some limnological differences, they are highly comparable because they are all endorheic, are located close to each other (< 20 km), and are influenced by similar climatic and edaphic conditions. Six of them are maar lakes, known as *axalapascos* (meaning bowls of sand filled with water) and were formed by different explosive eruptions caused by the contact between the ground water and magma during the Late Pleistocene, about 40,000 y.a. [38]. The other two are large, episodic playa lakes that fill during the rainy summer season (June-September). The *L*. cf. *sicilis* populations inhabit three maar lakes: Quechulac (19°22' N, 97°21’ W), La Preciosa (19°22′ N, 97°23′ W) and Atexcac (19°20′ N, 97°27′ W), which are permanent, deep (maximum depth, Z_max_ = 40, 45, and 39 m, respectively), warm monomictic (mixing occurs in winter), alkaline (pH 8.4–8.7), and oligotrophic lakes. Owing to the chemical composition of rocks and soil, they have distinct ionic compositions [37], but constant salinities along the annual cycle. Quechulac and La Preciosa are freshwater (Total Dissolved Solids, TDS = 0.42 ± 0.05 g L^-1^, specific conductivity at 25°C, K_25_– = 810 ± 25 μS cm^-1^, and TDS = 1.18 ± 0.09 g L^-1^, K_25_– = 2,220 ± 10 μS cm^-1^, respectively), while Atexcac is hyposaline (TDS = 6.54 ± 0.29 g L^-1^, K_25_ = 11,880 ± 62 μS cm^-1^). In addition, this copepod is also present in the playa lake El Carmen, also known as Laguna de Totolcingo (19°09' to 19°26' N, 97°33' to 97°47' W [39], which is shallow (< 30 cm), turbid, with variable salinity (TDS = 1.4–10 g L^-1^, K_25_ = 2,600–14,800 μS cm^-1^). In parenthesis are the average ± SD of 12 vertical profiles, measured monthly in 2009 at a fixed station located at the deepest part of each lake, except for El Carmen, where data are the range of 6 measures performed from September 2009 to February 2010, using a Hydrolab DataSonde 3/Surveyor 3 Multiparameter water quality logging systems (Hydrolab). A detailed description of the lakes can be found elsewhere [9,13,37,40–42].

### Sampling of copepods and culture conditions

Copepods were collected from all four lakes for morphological and molecular analyses, but fitness and reproductive tests were performed only with organisms from La Preciosa, Atexcac, and El Carmen. These lakes represent a freshwater stable environment, a brackish stable environment, and a variable ephemeral environment, respectively. Copepods were collected on September 2008 through vertical hauls with a conical zooplankton net (80 μm mesh size) in the crater lakes, or pouring water obtained with a bucket through the same net, in the playa lake. Fractions of the sample were fixed with formalin (4% final concentration) or with 100% ethanol, for the morphological and the molecular analyses. Some organisms were kept alive and transported to the laboratory. Adult ovigerous females were isolated and cultured in 4 L glass jars at the salinity recorded at the time of collection (La Preciosa: 1.1 g L^-1^, Atexcac: 6.5 g L^-1^ and El Carmen: 3.8 g L^-1^). Cultures were maintained at 18±1° C in a photoperiod (12:12, light:dark) and fed with the microalgae *Tetraselmis suecica* and *Chlorella vulgaris* (1:1; >20 mg C L^-1^) during at least 2 months before starting the experiments (approx. two acclimation generations). The culture medium was prepared with commercial salt (Seachem Reef Salt, Seachem Laboratories, U.S.A.) dissolved in electrodeionized (Millipore, Elix-5) that had been previously autoclaved at 121° C for 15 min. *T*. *suecica* was cultured in saline medium (18 g L^-1^), while *C*. *vulgaris* was cultured in EPA medium [43], both enriched with *f*/2 modified medium [44].

### Morphological analysis

Our analysis involved whole and dissected adult organisms of both sexes, using standard procedures in the taxonomy of the genus *Leptodiaptomus* [45,46]. A Leica DM LB2 compound microscope with a drawing tube was used for observations, measurements and drawings of dissected specimens at 1000×. Other individuals were observed with scanning electron microscopy (SEM) using a Hitachi S-400 and a JEOL JSM6360LV microscopes. We analyzed in detail those characteristics that define *L*. *sicilis* s.s. [26] as a taxonomic species, with emphasis on the structures involved in sexual recognition and mating [47,48]. Additionally, we examined body size (total length excluding the caudal ramus), color (pigmentation), and clutch size (number of eggs per sac). To describe differences in the size of referred structures between populations, a one-way ANOVA was performed and a Student-Newman-Keuls *post hoc* test (SNK) [49] was carried out if significant differences were found (*P* < 0.05). Also, to determine differences between relative body sizes in both males and females from each population, data were analyzed using the non-parametric test of Kruskal Wallis [50]. If differences among lakes were found, pairwise Mann-Whitney U tests were carried out (*P* > 0.05). All statistical analyses were made with SPSS 17.0 software (SPSS Inc., Chicago).

To compare individuals from Mexican populations to individuals from areas near the type locality (Lake Michigan, U.S.A.) where *L*. *sicilis* was described, we analyzed zooplankton samples collected and donated by Dr. Manuel Elías-Gutiérrez (El Colegio de la Frontera Sur, Mexico) from lakes Erie (Collected at Erie, Pennsylvania, 29-Jun-10, Lorain, Ohio, 2-Jul-10 and Toledo, Ohio, 10-Jul-10), Huron (Warden, Ontario, 25-Jul-10; Wiarton, Ontario 2, 25-Jul-10) and Detroit River (Windsor, Ontario, 30-Jul-10). Unfortunately, we did not find any *L*. *sicilis* individuals, because they were either scarce or absent at the sampling locations/dates, and sampling was not exhaustive.

### COI sequencing and genetic divergence analyses

Sequence variation in mtDNA COI was assessed in the four populations of *L*. cf. *sicilis* by isolating adult copepods from the ethanol-fixed samples. DNA was extracted by the HotSHOT method, after which COI was amplified using PCR methods described by Montero-Pau *et al*. [51]. Each PCR reaction had a total volume of 50 μL and contained 35.2 μL of ultrapure water, 5.0 μL of 10x PCR Buffer, 1.5 μl of MgCl_2_, 1.0 μL of each LCO1490 and HCO2198 primers [52], 1.0 μL of dNTP's, 0.3 μL of *Taq* polymerase (1.5U) and 5.0 μL of DNA template. 4 μL of PCR product were separated by electrophoresis in 50x TAE buffer in a 1% agarose gel and visualized with UV-light fluorescence. PCR products were sequenced bidirectionally using an ABI 3130 capillary sequencer with BigDye Terminator v.3.1 [53]. Electropherograms were analyzed and edited with Chromas 2.13 (Technelysium Pty Ltd., Queensland). Sequences of closely related copepod species obtained from the Barcode of LifeData Systems (www.boldsystems.org) were also included for comparison. The species were (accession numbers): *L*. *siciloides* (ZPLMX814-06, ZPLMX816-06 and ZPLMX817-06), *L*. *minutus* (EU825134, EU825137 and EU825188), *L*. *novamexicanus* (ZPLMX182-06, ZPLMX921-06 and ZPLMX922-06), *L*. *garciai* (ZPII068-07, ZPII074-07, and ZPII076-07) and *L*. *cuauhtemoci* (ZPII1346-11, ZPII1360-11 and ZPII1196-11). *Mastigodiaptomus albuquerquensis* (ZPLMX248-06, ZPLMX526-06 and ZPLMX528-06) was the external group. *L*. *sicilis* s.s. sequences were not included because they are not available from BOLD Systems or GenBank.

For phylogenetic analysis, sequences were aligned using ClustalW in MEGA 5 software [54]. Genetic distances were calculated using the Kimura two-parameter (K2P) distance model [55,56]. Neighbor-joining trees using K2P distances [57] generated a graphical representation of divergence pattern between the Oriental Basin populations and related species. Using MEGA 5 we identified the degree of variation between sequences, nucleotide diversity and the proportion of variable sites (*P* distance). Different haplotypes in populations, polymorphic sites, nucleotide (π) and haplotype (H_D_) diversities were detected with DNASP 4.1 [58]. Fixation indices (*F*_ST_) were calculated with ARLEQUIN 3.11 [59] to estimate the degree of differentiation between populations; AMOVA was performed to calculate intra and inter-population variation. A haplotype network was constructed using the statistical parsimony method with ANeCA v.1.2 [60], which includes TCS 1.21 [61] and GeoDis 2.5 [62]. Clades were nested according to Templeton *et al*. [63] criteria. Finally, the relationship between genetic variation and geographic location was tested with GeoDis 2.5 while the different haplotype patterns (allopatric fragmentation, range expansion or isolation by distance) were analyzed using the inference key of Posada and Templeton [64].

### Life history variation in reciprocal transplant experiments

#### Hatching of resting eggs from lake sediments

To estimate the viability of resting stages of *L*. cf. *sicilis* as effective dispersal agents among lakes (passively dispersed by wind or waterfowl), we analyzed the hatching of resting eggs at three salinities comprising the range of salinity recorded in the lakes: Eggs from La Preciosa, Quechulac and Atexcac were tested at 1.1 g L^-1^, 6.5 g L^-1^ and 9 g L^-1^, eggs from El Carmen were tested at 1.1 g L^-1^, 3.8 g L^-1^ and 9 g L^-1^. Quantitative samples of superficial sediment (top 3 cm) were collected from the deepest part of Atexcac, La Preciosa and Quechulac with an Eckman dredge (area = 0.0625 m^2^), and a sample of mixed sediments obtained from several places at the shore of El Carmen. Sampling of the eggs contained in the superficial sediment layer allowed us to obtain a representative sample of genotypes produced during several years (at least 20 y at each lake if calculated on the sedimentation rate of 0.16 cm yr^-1^ measured in the neighboring Lake Alchichica [65]). Sediments were transported to the laboratory in total darkness at 4° C [66]. Experimental units consisted of glass flasks with 3 cm^3^ of sediment and 20 ml of culture medium, placed inside an environmental chamber at constant conditions of temperature, photoperiod, and light intensity (18 ±1° C, 13 hours light, and 280 mol quanta m^-2^ s^-1^). Six replicates were performed for a total of 54 experimental units (3 populations × 3 salinities × 6 replicates). Daily observations and counts of hatched eggs were made for 25 days. As the density of resting eggs in the sediments was unknown, we calculated the accumulated hatchings as the percentage of the mean value of the highest numbers of individuals hatched from a replicate of the six performed for each salinity treatment per studied lake (*n* = 3). We compared the accumulated hatching during the last 5 days of the experiment using Generalized Linear Models (GLM) [67] with a binomial distribution and a link logit function carried out using *R* [68]; salinity and population were considered as fixed factors.

#### Survivorship and development

A reciprocal transplant experiment was performed to analyze the survivorship and development of La Preciosa, Atexcac and El Carmen populations at the salinities recorded in the wild at the time of collections (1.1, 3.8 and 6.5 g L^-1^), according to the methods proposed by Montiel-Martínez *et al*. [13]. To reduce the risk of osmotic shock, all copepods were gradually acclimated to the experimental salinities before starting the experiment, transferring them every 24 h along a gradient of increasing or decreasing salinities during five days (e.g., 1.1–2.5–3.8–5.1–6.5 g L^-1^ in La Preciosa copepods). CIII copepods were chosen as experimental subjects because in pre-experimental cultures, mortality rates at earlier developmental stages were highly variable among populations, even at their native salinity conditions. Copepods were individually placed into wells of polystyrene plates (six wells each) containing 8 mL of medium and abundant food (*T*. *suecica* and *C*. *vulgaris* at 1:1; >20 mg C L^-1^); 8 replicates (i.e., a plate with six individuals) were performed per salinity treatment and population (6 individuals × 8 plates × 3 salinities = 144 individuals per population). Experimental units were examined daily under a stereomicroscope Leica MZ95 to record survival and molting, and then copepods were transferred to new plates with fresh medium and food. Survival curves obtained after at least 15 days of observations were analyzed using the Kaplan-Meier method [69], and pairwise comparisons (*P*≤0.05) using Log-rank tests [70]. Time (in days) to molt from CIII to adult was analyzed to determine the effect of salinity and population using two-way ANOVAs [49]. If significant differences were found (*P* ≤ 0.05), *post hoc* SNK tests were carried out.

#### Intrapopulation mating success

Mating trials were conducted to evaluate the effect of salinity (1.1, 3.8 and 6.5 g L^-1^) on the reproduction of La Preciosa, El Carmen, and Atexcac populations. Males and females were used in a 2:1 ratio to increase the possibility of a successful mating [13]. The organisms were selected from the pre-experimental cultures, choosing pre-adult females (CV stage) to ensure they were unmated. Twelve triads from each lake were placed separately in 50 ml flasks with medium at the three different salinities, plus food (3 populations × 12 triads × 3 salinities). Observations and medium renewal were performed daily for at least 15 days. Dead males were removed and replaced. Females that had copulated (identified by the presence of at least one spermatophore) were individually transferred to fresh medium and maintained in the experimental conditions until the appearance of the egg sac and the hatching of larvae. For each combination of population and salinity we calculated the percentage of females that copulated, the egg ratio (number of eggs produced/ total number of females), hatching success (hatched larvae/eggs), and the relative hatching (hatched larvae/ total number of females) [13,71,72]. The effect of salinity and population were analyzed using non-parametric Scheirer–Ray–Hare tests (SRH) [50].

We interpreted the ANOVA, GLM and SRH results considering that (1) a significant effect of salinity is evidence for overall plasticity, (2) a significant effect of population is evidence for genetic clustering, and (3) a significant interaction between the two factors is evidence of genetic differentiation among populations in plasticity [9,73]. Accordingly, local adaptation occurs if the interaction between salinity and population is significant and a population shows higher resting egg hatching, faster development or a better reproductive success in its local salinity than populations from the other lakes (the ‘local versus foreign’ criterion [74]). Thus, this criterion was useful in identifying selection against migrants, namely, when the environment disfavors migrants relative to natives [75], the quotient fitness of migrants/fitness of residents < 1. We also calculated the relative fitness of organisms in the two alternate salinities (fitness in alternate salinity/fitness in native condition) as a measure of the cost for dispersal to environments of different salinity.

### Interpopulation mating success

This analysis was performed to test for premating and postmating barriers among the populations from the Oriental Basin. We compared mating success between males and females from different lakes and compared them with those obtained in the intrapopulation breeding experiment. The experimental salinity for the common garden experiment was 3.8 g L^-1^ because that was the condition at which the three populations showed similar copulation rates (the extreme salinities reduced considerably the fitness of La Preciosa and Atexcac populations). Copepods were taken from pre-experimental cultures and were acclimated gradually to achieve the final experimental condition. We used ‘no choice’ mate tests [76], where males within each triad belonged to one population while the females were from another. Each experimental unit consisted of a female plus two males in a flask with 50 ml of medium with abundant food and controlled abiotic conditions. We performed all the crosses (Table 1) excepting Atexcac females *×* El Carmen males because pre-experimental organisms from El Carmen died prior to obtaining the experimental males. Overall, we performed 5 inter-population trials (10 replicates each) and used the intrapopulation crosses at 3.8 g L^-1^ as controls. If inter-population copulation occurred, females were individually isolated to observe egg production and larvae hatching as in intra-population trials. Results were analyzed for the effect of the origin of male and female using non-parametric SRH tests in order to determine significant differences among crosses.

**Table 1:** Mating trials performed for fitness and reproductive compatibility assessment.

	♂ Atexcac	♂ La Preciosa	♂ El Carmen
♀ Atexcac	*i*	×	NO
♀ La Preciosa	×	*i*	×
♀ El Carmen	×	×	*i*

Sex of individuals and lake of origin are shown. *i*: intra-population crosses; ×: inter-population crosses; NO: not performed (consult the text for details).

## Results

### Morphological divergence

After comparing individuals from the four populations with the original description of Forbes [26], and with other identification keys and specialized literature [77–82], we found that all specimens corresponded well with the diagnostic description of *Leptodiaptomus sicilis*. Detailed analysis based on dissections, drawings, photographs, and direct observations of numerous individuals with compound and scanning electron microscopes revealed no significant differences in the morphology of structures involved in mate recognition or in the presence or absence of spines, processes or membranes used for taxonomic purposes. Nevertheless, there were significant differences in body length in both males and females (Kruskal-Wallis; *P* ≤ 0.05); each population constituted a distinct group with minimal overlap: El Carmen > Quechulac > La Preciosa > Atexcac (Table 2A). Males were about 10% smaller than females in the four populations. Contrasting pigmentation among populations was also observed: red color in El Carmen and Atexcac, and colorless in Quechulac and La Preciosa. The characteristic pigmentation observed in field populations was persistent in the laboratory cultures maintained for several months.

**Table 2:** Adult body size (mm) of individuals from *Leptodiaptomus sicilis* and *L*. cf. *sicilis*.

	Lake	Sex	Range (mm)	Mean ± S.E.	Relative size ♂ / ♀
A	Atexcac	♀	0.86–0.90	0.88 ± 0.003^a^	0.88
		♂	0.75–0.81	0.78 ± 0.003^w^	
	La Preciosa	♀	1.03–1.13	1.10 ± 0.006^b^	0.85
		♂	0.89–0.99	0.94 ± 0.006^x^	
	Quechulac	♀	1.21–1.37	1.27 ± 0.012^c^	0.83
		♂	1.01–1.12	1.27 ± 0.007^y^	
	El Carmen	♀	1.29–1.39	1.34 ± 0.007^d^	0.89
		♂	1.16–1.26	1.20 ± 0.008c^z^	
B	Michigan	♀	1.2–1.3	1.25	0.88
		♂	1.0–1.2	1.10	
	Erie	♀	1.2–1.9	1.55	0.84
		♂	1.1–1.5	1.30	
	Superior	♀	1.5–1.8	1.66	0.83
		♂	1.3–1.4	1.38	
	Oriental Basin	♀	0.9–1.4	1.14	0.87
		♂	0.8–1.3	0.99	

(A) Populations of *L*. cf. *sicilis* from Oriental Basin, Mexico. Different letters indicate significant differences among females (^a, b, c, d^) and males (^w, x, y, z^) of the four populations, according to Mann-Whitney U tests (*P* < 0.05; *n* = 20, for each sex and population); (B) Populations from the Great Lakes, USA (see text for references) and Oriental Basin, Mexico (average, this study).

### COI sequencing and genetic divergence

**Fig 1:**
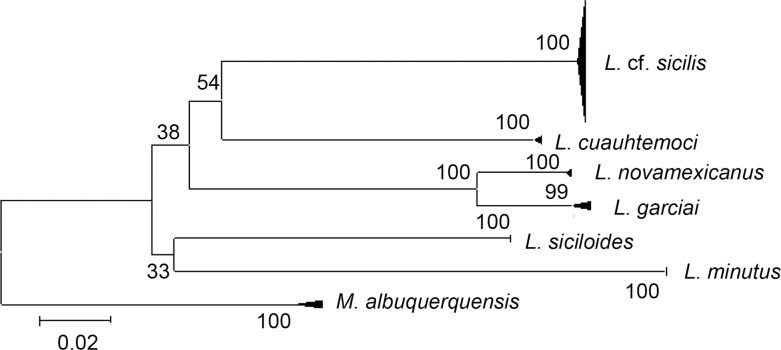
Simplified neighbor-joining tree of COI sequences. The branch of *L*e*ptodiaptomus* cf. *sicilis* comprises individuals from Atexcac, La Preciosa, El Carmen and Quechulac lakes (Oriental Basin) and is compared to congeneric species using K2P genetic distances. *Mastigodiaptomus albuquerquensis* was used as outgroup species. Numbers over branches indicate percent bootstrap support (1000 replicates).

**Fig 2:**
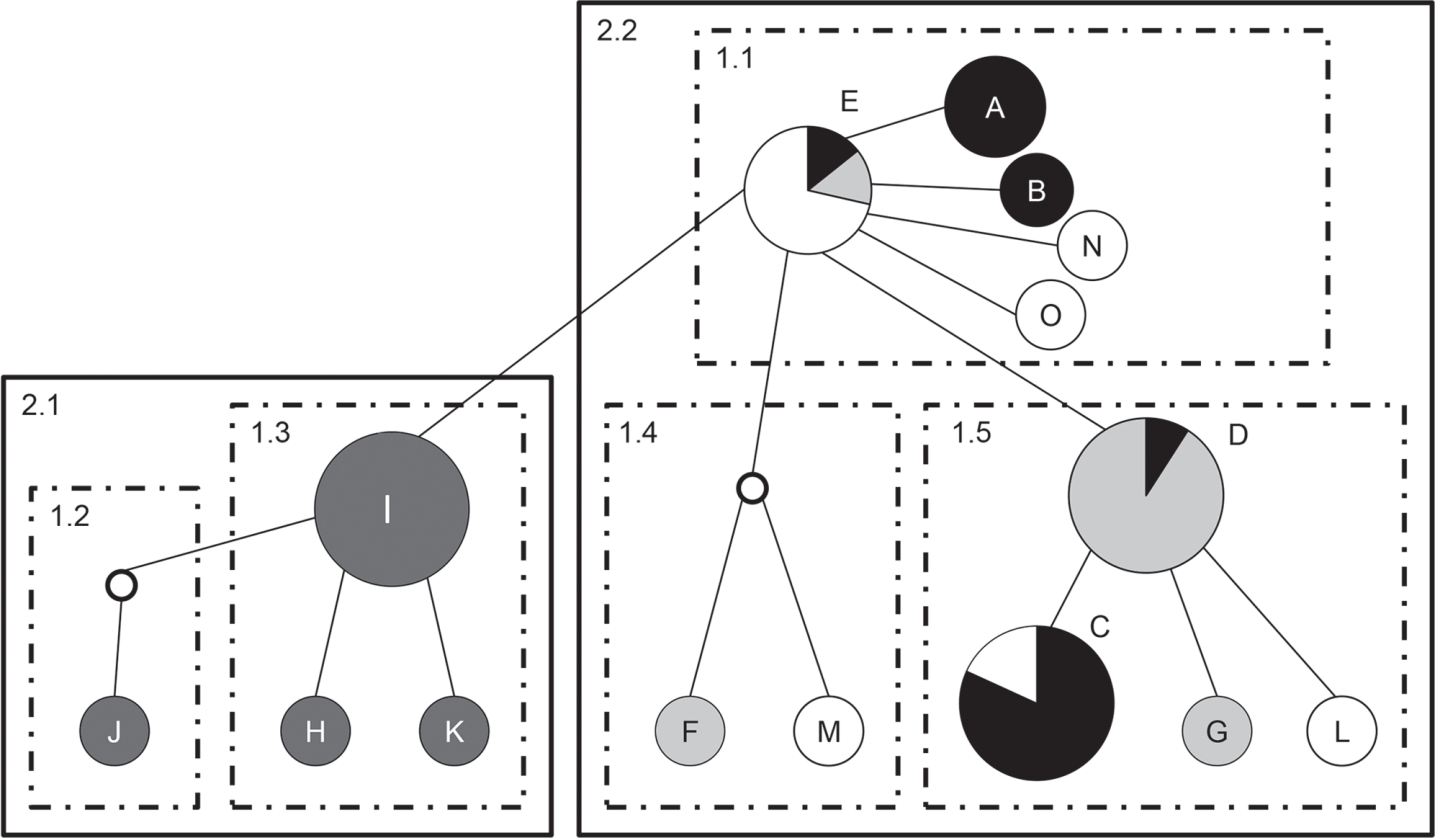
Nested clade design for the haplotype network of COI sequences of *Leptodiaptomus* cf. *sicilis* populations. A-O: Haplotypes found. Circle size represents haplotype frequency; color indicates the lake where haploptypes are distributed. Black: El Carmen; dark gray: Atexcac; light gray: La Preciosa; white: Quechulac. Haplotype (E) corresponding to the most extended and probably ancient one. White, unlabeled circles represent inferred haplotypes. Informative clades are indicated with numbers (*x*.*y*).

We obtained a total of 48 COI sequences from the four populations studied (Atexcac = 12; La Preciosa = 12; El Carmen = 13; Quechulac = 11). The GenBank accession numbers are KP213127—KP213174. The alignment included 652 bp; other sequences were too short for comparison (<550 bp). The mean values of genetic distances generated by the K2P model are summarized in Table 3, the greatest divergences were found between Atexcac and the other populations: 0.43% with El Carmen, 0.37% with La Preciosa, and 0.35% with Quechulac. A clear divergence with respect to the other *Leptodiaptomus* species included in the comparison (> 20%) was revealed in the simplified consensus identity NJ tree based in the K2P genetic distances (Fig 1). The sequence alignment revealed 16 polymorphic sites (nucleotide diversity π = 0.0028). 15 different haplotypes were obtained from the four populations with a haplotype diversity H_D_ = 0.875. Only three haplotypes were present in more than one lake: Haplotype E (Fig 2) is shared among El Carmen, La Preciosa and Quechulac, haplotype C is shared between El Carmen and Quechulac, and haplotype D was found in copepods from El Carmen and La Preciosa. Contrastingly, in each lake there were 2–4 unshared haplotypes, and the set of four present in Atexcac were absent in the other lakes. The fixation index *F*_ST_ between populations (0.113–0.697; *P* ≤ 0.05) indicated the absence of recent gene flow among the four populations. The AMOVA revealed significant intrapopulation variation (57.2%; *P* ≤ 0.05). The nested clades of the haplotype network showed five first-level and two second-level clades, one of which (2.1) correspond to the Atexcac population. Finally, the inference key [64] indicated a pattern of restricted gene flow/dispersal with some long-distance dispersal within the clade 2.2 (haplotypes from El Carmen, La Preciosa and Quechulac), and a pattern of allopatric fragmentation among 2.1 and 2.2 clades.

**Table 3:** Genetic divergence (mean ± SE) between distinct clades of *L*. cf. *sicilis* populations and other *Leptodiaptomus* species.

	Car	Pre	Ate	Que	Ls	Ln	Lm	Lg	Lc	Ma
Car	0.24 ± 0.1									
Pre	0.23 ± 0.1	0.12 ± 0.1								
Ate	0.43 ± 0.2	0.37 ± 0.2	0.10 ± 0.0							
Que	0.28 ± 0.1	0.23±0.1	0.35 ± 0.2	0.26 ± 0.1						
Ls	22.5 ± 2.2	22.5 ± 2.3	22.5 ± 2.3	22.5 ± 2.3	0.0 ± 0.0					
Ln	22.1 ± 2.3	22.0 ± 2.3	22.0 ± 2.3	22.0 ± 2.3	20.1 ± 2.2	0.13 ± 0.1				
Lm	26.8 ± 2.6	26.8 ± 2.6	26.8 ± 2.6	26.9 ± 2.6	23.4 ± 2.3	25.2 ± 2.5	0.0 ± 0.0			
Lg	22.7 ± 2.4	22.6 ± 2.4	22.6 ± 2.3	22.7 ± 2.4	20.3 ± 2.1	5.90 ± 1.1	25.5 ± 2.5	0.77±0.3		
Lc	19.4±2.1	19.3±2.1	19.4±2.1	19.4±2.0	19.9±2.1	19.1±2.0	25.0±2.5	20.4±2.2	0.39±0.2	
Ma	25.3 ± 2.5	25.4 ± 2.4	25.4 ± 2.5	25.3 ± 2.4	22.6 ± 2.3	27.0 ± 2.6	28.9 ± 2.8	27.3 ± 2.6	27.4±2.6	1.0 ± 0.4

Distances are Kimura-2-parameter distance (%), with diagonal values indicating intra-clade genetic variation. Clades are shown in Fig 1. Car: El Carmen; Pre: La Preciosa; Ate: Atexcac; Que: Quechulac; Ls: *L*. *siciloides*; Ln: *L*. *novamexicanus*; Lm: *L*. *minutus*; Lg: *L*. *garciai*; Lc: *L*. *cuauhtemoci*; Ma: *M*. *albuquerquensis*.

### Comparison of performance at different salinities

#### Hatching from resting eggs

**Fig 3:**
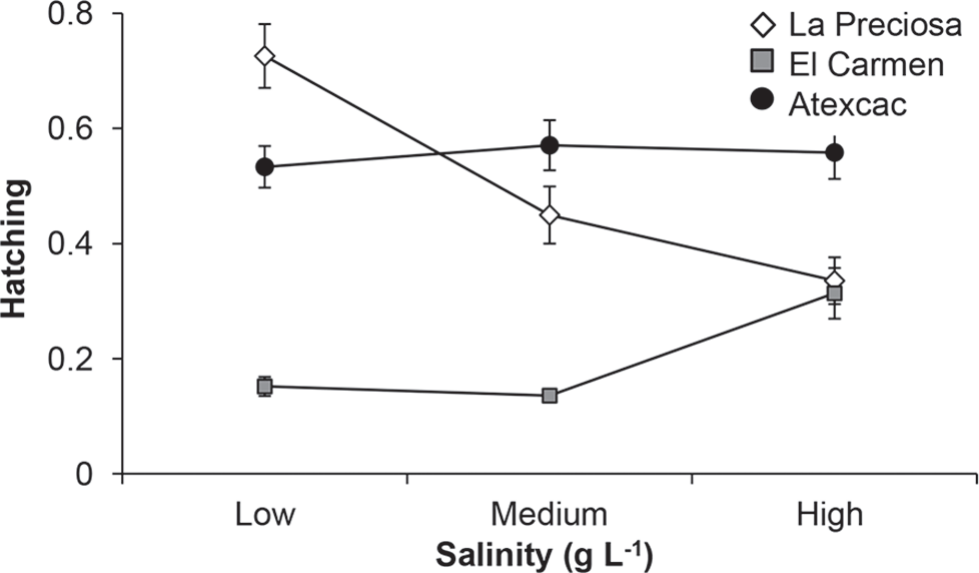
Reaction norms for the proportion of hatching in resting eggs from three *L*. cf. *sicilis* populations at several salinities. Low: 1.1 g L^-1^; Medium: 6.5 g L^-1^ for La Preciosa and Atexcac, 3.8 g L^-1^ for El Carmen; High: 9.0 g L^-1^.

No resting eggs of *Leptodiaptomus* were found or hatched from sediments of Quechulac. The reaction norms and statistical analyses of the maximum hatching attained (Fig 3 and S1 Table) indicate that salinity had a significant effect on resting egg hatching, as well as a significant salinity × population interaction (GLM test; *P* < 0.05). The absolute highest hatching percentage was attained by eggs from La Preciosa at their native salinity (1.1 g L^-1^); this was significantly higher than hatching of other populations at that salinity. The response of eggs from Atexcac did not change significantly at the tested salinities, but the performance of this population was the best (compared to La Preciosa) at its local salinity (6.5 g L^-1^). Resting eggs from La Preciosa decreased significantly in their performance in the two higher salinities, whereas eggs from El Carmen were negatively affected by freshwater.

#### Survivorship and development

**Fig 4:**
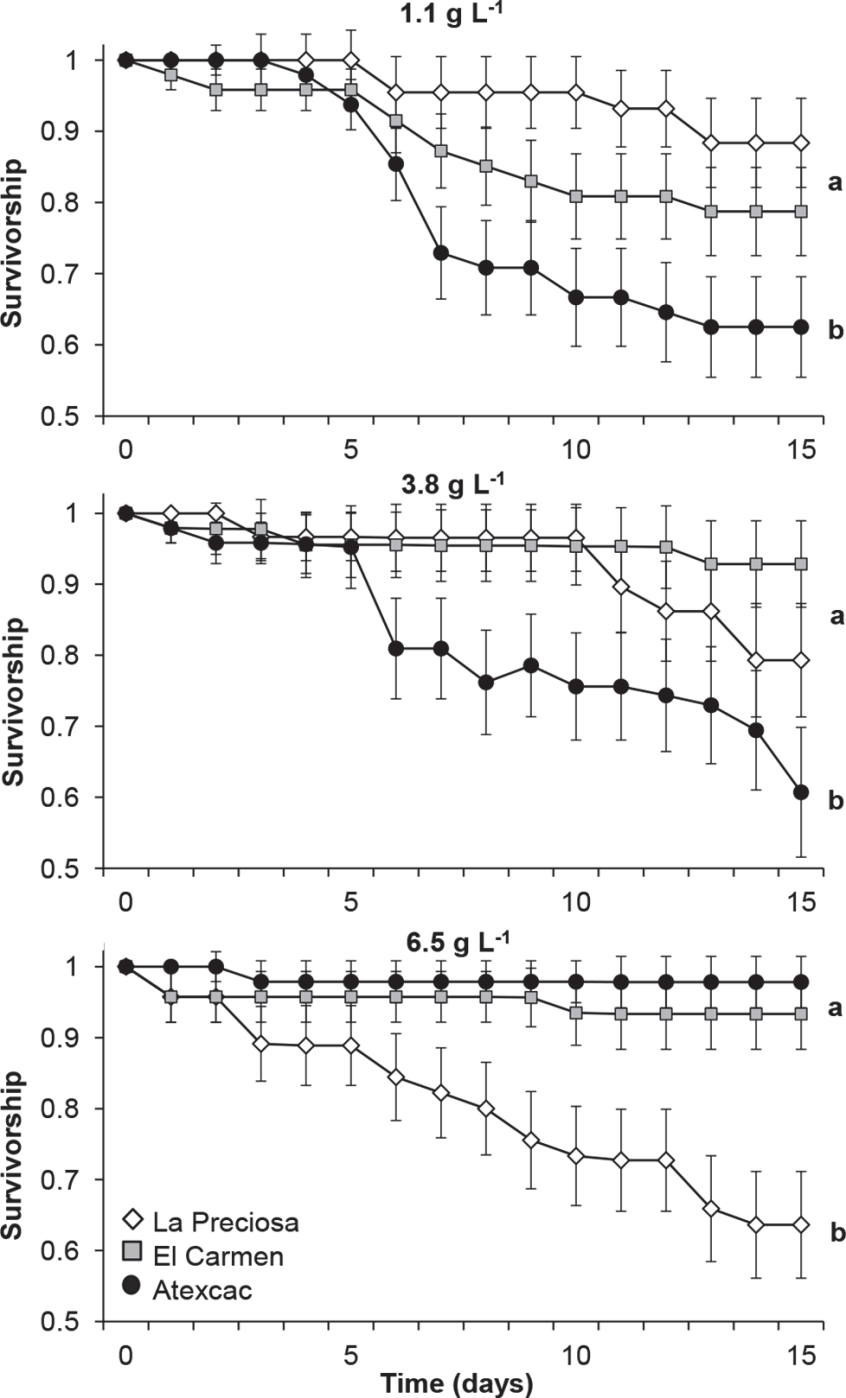
Survivorship curves of three populations of *L. cf. sicilis* at home and alternative salinities. Values are means ± SE; letters indicate significant differences among populations according to the log-rank tests (*P* ≤ 0.05).

**Fig 5:**
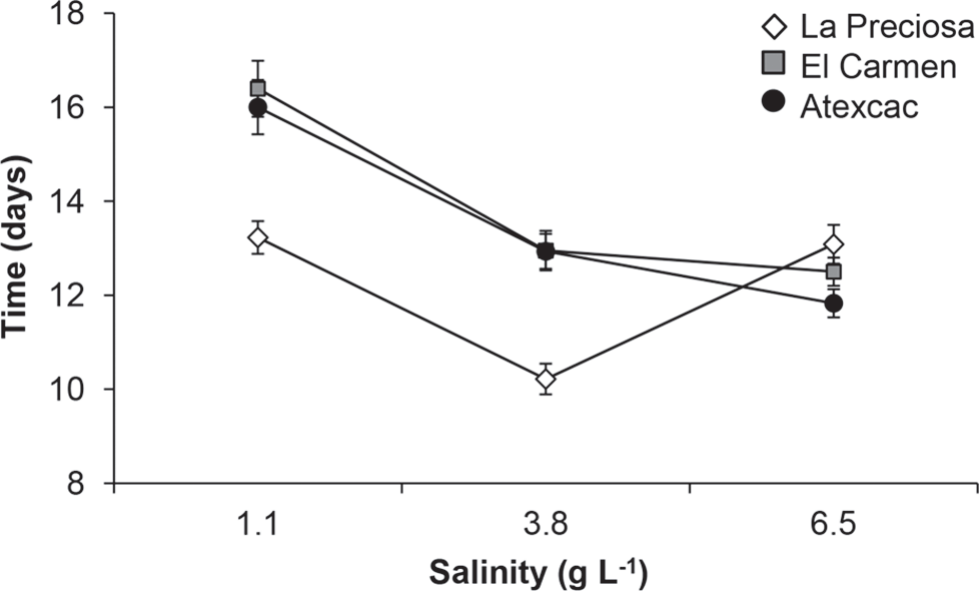
Development (time to molting from CIII to adult stage) of the three studied populations of *L*. cf. *sicilis* at home and alternate salinities. Values are means ± SE.

We observed that the three populations had differential responses to salinity, with general effects of salinity and population as individual factors (Figs 4 and 5). Copepods from La Preciosa and Atexcac had the highest survival rate at their native salinity (1.1 g L^-1^ and 6.5 g L^-1^, respectively) and were negatively affected by increased or decreased salinity. Performance of individuals from El Carmen was statistically similar at the three salinities, comparable to La Preciosa at 1.1 and 3.8 g L^-1^, and to Atexcac at 6.5 g L^-1^ (Log-rank tests; *P* < 0.05). Thus, the clearest divergence in survival was between La Preciosa and Atexcac.

Salinity and population showed significant effects on development time of individuals from CIII to adult stage; in addition, the salinity × population interaction was also significant (two-way ANOVA, *P* < 0.001; Fig 5). The mean development time was longest at the lowest salinity. Copepods from La Preciosa and Atexcac reached the adult stage faster than El Carmen. Individuals from La Preciosa and (to a lesser degree) Atexcac developed more rapidly at their native salinities compared to the other populations. When transplanted to fresh water, the development of individuals from El Carmen and Atexcac was slower.

#### Intra-population mating

**Fig 6:**
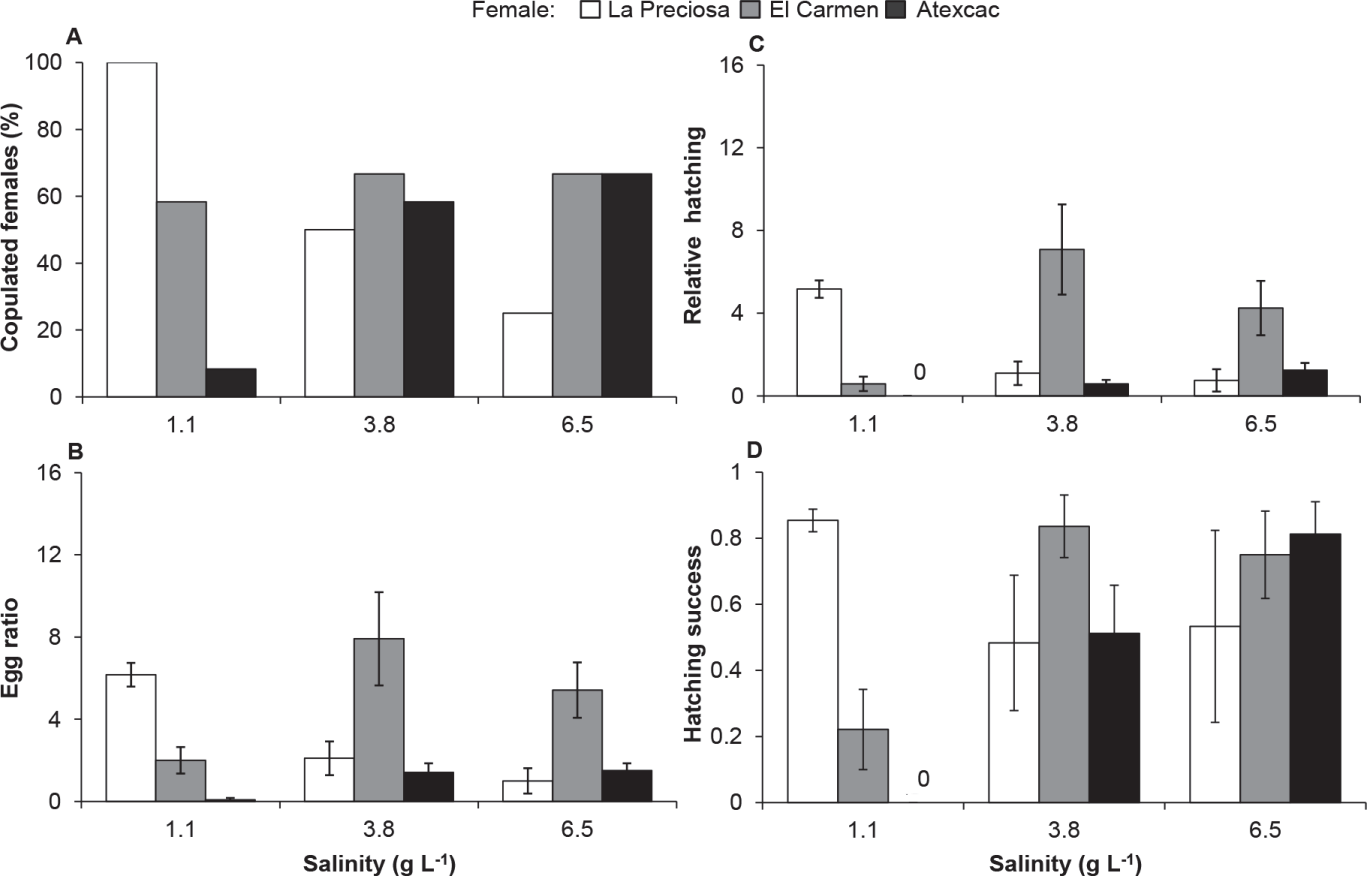
Intra-population mating at home and alternative salinities. (A) Percentage of copulated females (spermatophore attached to the genital pore). (B) Egg ratio (number of eggs/number of females); (C) Relative hatching (number of nauplii/number of females); (D) Hatching success (nauplii/eggs). Values are mean ± SE.

The proportion of copulated females was on average higher in El Carmen (64%) and La Preciosa (58%) and lower in Atexcac (44%). Salinity had important effects on the number of copulated females in La Preciosa and Atexcac populations, whereas the effect was minor in El Carmen (Fig 6A). Animals from La Preciosa reached the highest number of copulated females at their native salinity (1.1 g L^-1^), and decreased noticeably at increased salinities. Contrastingly, the lowest salinity had an important negative effect on the number of copulated females (Fig 6A) in copepods from Atexcac; these attained their highest copulation rate at the salinity of their natural habitat (6.5 g L^-1^).

Among mated females, mean clutch size (eggs per egg sac) at the native salinity was different for each population: El Carmen (11.9 ± 6.6 SD) > La Preciosa (6.2 ± 2.0 SD) > Atexcac (2.3 ± 0.7 SD). Correspondingly, only the effect of population and salinity × population (S×P) interaction were significant on egg ratio and relative hatching. On the other hand, only S×P interaction was significant on hatching success (SRH tests, *P* < 0.001; S2 Table). The decreasing order in clutch size from El Carmen to Atexcac was also mirrored in egg ratio and relative hatching, with the best performance of El Carmen at 3.8 g L^-1^ (Fig 6B, 6C and 6D). In their native conditions, females from La Preciosa and El Carmen (1.1 and 3.8 g L^-1^, respectively) performed better than the other populations in all parameters (Fig 6A–6D), but their performance declined throughout the salinity gradient. Animals from Atexcac were negatively affected by alternative salinities and their performance at native salinity was not the best compared to foreign organisms.

**Fig 7:**
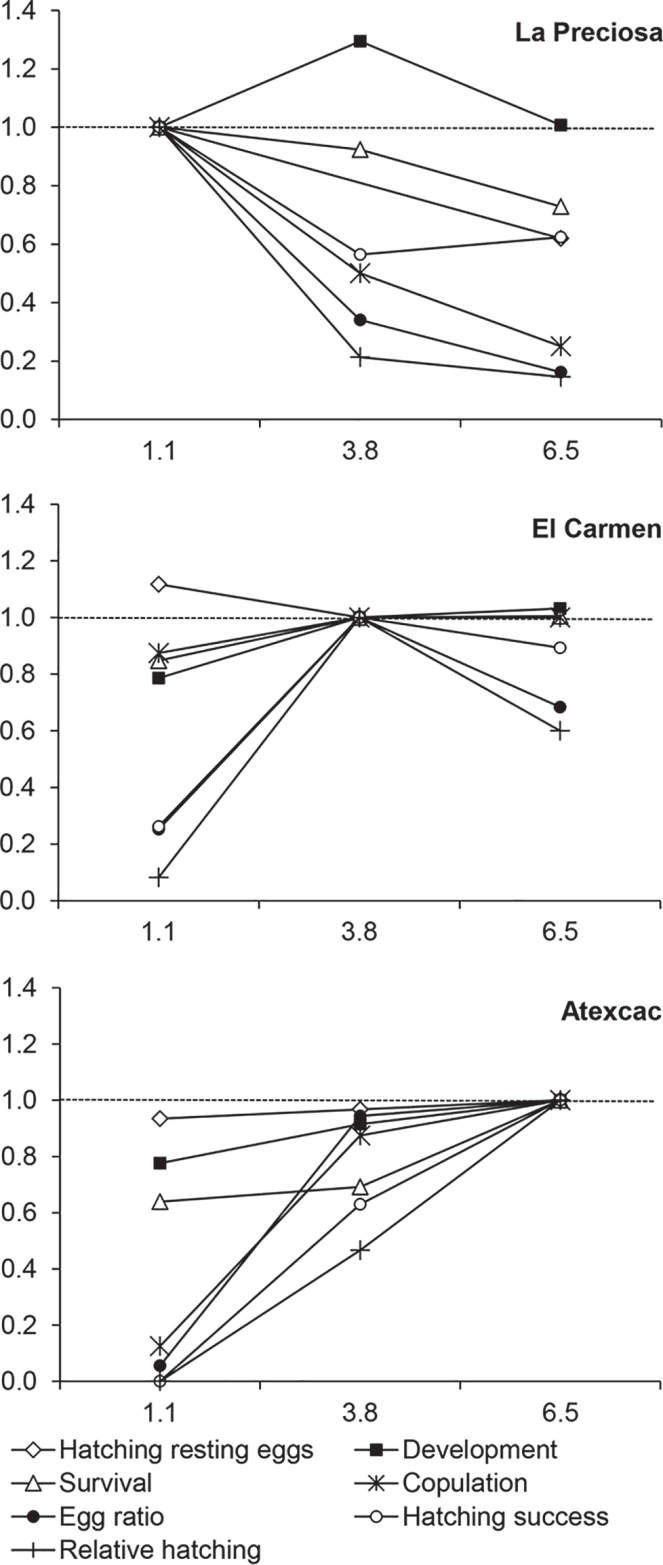
Performance of populations of *L*. cf. *sicilis* at alternate salinities relative to home conditions. Fitness at home conditions = 1, pointed line.

Overall, relative fitness of organisms from La Preciosa decreased at the higher salinities of the other lakes, most remarkably in the egg ratio, copulation, and hatching of diapausing eggs, and to a lesser degree on survival (Fig 7). Alternate environments also negatively affected individuals from Atexcac, with dramatic effects of freshwater on egg ratio, hatching of subitaneous eggs (eggs that develop immediately without a period of dormancy), and copulation, although development and survival decreased too. Only the hatching of resting eggs was unaltered. Regarding the population from El Carmen, fresh water had a pronounced negative impact on the six variables considered (except for the resting egg hatching), but the highest salinity also decreased the egg ratio and the hatching of subitaneous eggs.

**Fig 8:**
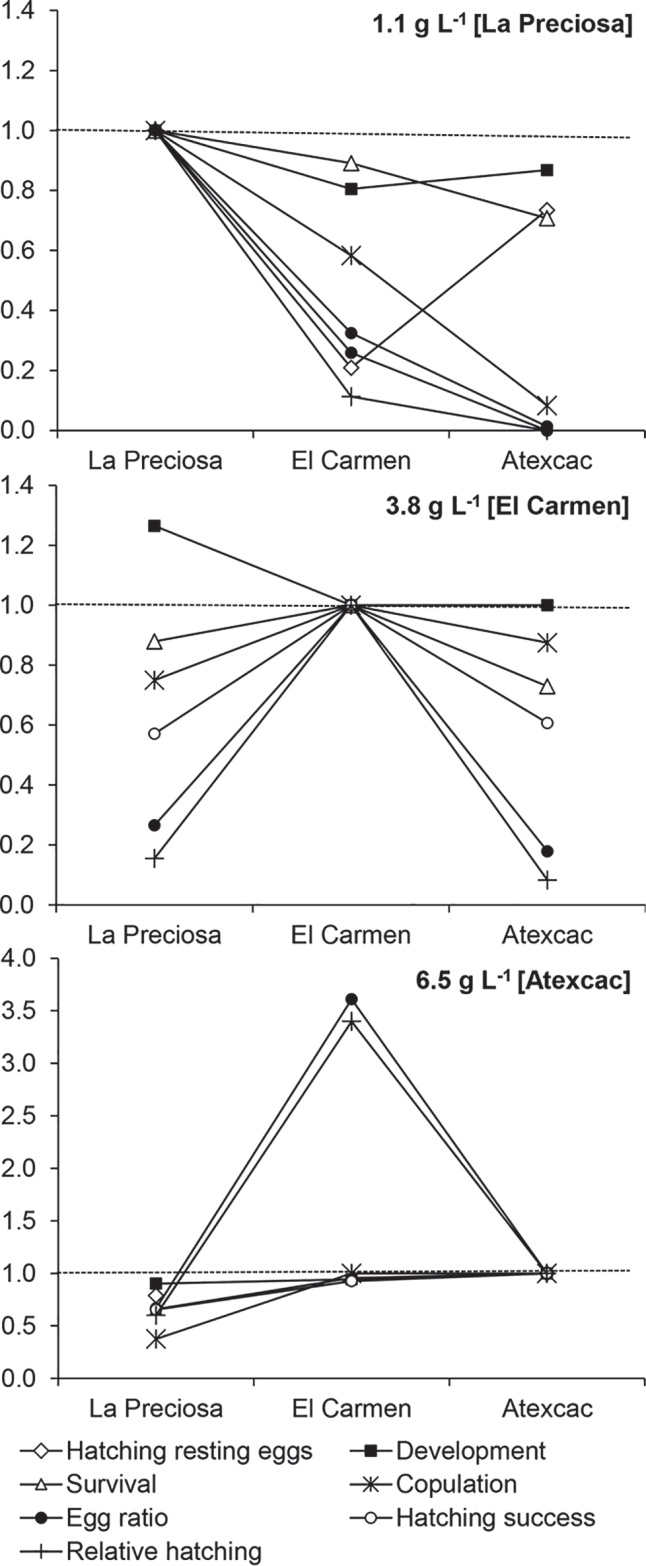
Selection against immigrants. Performance of immigrants from populations of *L*. cf. *sicilis* in different salinities relative to resident populations. Resident population between brackets. Fitness of resident populations = 1, pointed line.

In the test for selection against migrants (Fig 8), we found a decreased fitness of immigrants from El Carmen and Atexcac at Lake La Preciosa (1.1 g L^-1^) compared to the resident population. The negative effects were more dramatic on copulation, egg ratio, and hatching of subitaneous eggs from Atexcac. At the salinity of lake El Carmen (3.8 g L^-1^), the performance of immigrants from the other two lakes was inferior to the resident population except in development rate, with La Preciosa performing better than the local population. Finally, at the salinity of Lake Atexcac (6.5 g L^-1^), fitness of immigrants from La Preciosa was lower than the resident population, but hatching success and development were only slightly inferior (0.90 of the local population for both variables). The performance of individuals from El Carmen was very similar to the resident population, and even the egg ratio and relative hatching exceeded almost 4 times the observed on local females.

### Reproductive compatibility

#### Inter-population breeding

**Fig 9:**
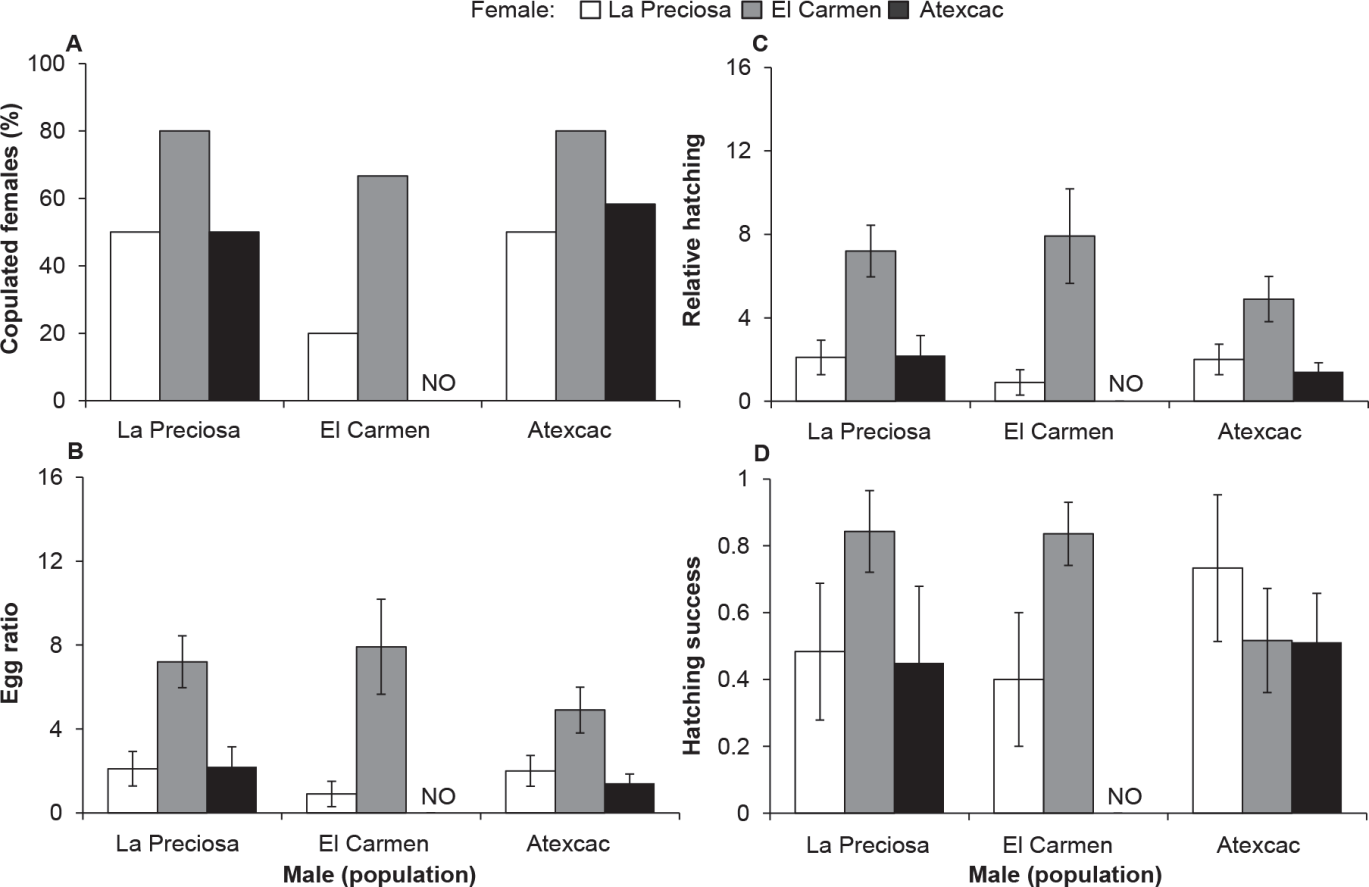
Reproductive compatibility among populations in a common garden experiment (3.8 g L^-1^). (A) Percentage of copulated females (spermatophore attached to the genital pore). (B) Egg ratio (number of eggs/number of females); (C) Relative hatching (number of nauplii/number of females); (D) Hatching success (nauplii/eggs). Values are mean ± SE.

Recognition of mates, attachment of spermatophores by males to females (Fig 9A), egg formation (Fig 9B), and hatching of nauplii (Fig 9C and 9D) occurred in all interpopulation crosses performed at an intermediate salinity (3.8 g L^-1^), although significant differences existed due to female origin in egg ratio and relative hatching (S3 Table; *P* < 0.005). Females from El Carmen were more frequently copulated by males from the three populations, at percentages even higher (80%) than in interpopulation crosses (65%) (Fig 6). Also, females from El Carmen showed higher egg ratio and relative hatching than the other two populations, which had similar performances.

## Discussion

### Morphological and genetic divergence

Copepods from our four study lakes showed significant differences among populations in body size and pigmentation. However, no differences were observed in the size or shape of the characters analyzed, particularly those involved in the reproduction, e.g., geniculate antennule, claw in P5 of males, or structure of P5 in both sexes. Thus, body size could be the only factor that interferes in sexual recognition or compatibility during interpopulation mating and constitute a barrier to gene flow (but see results on interbreeding trials below).

Because the animals used for morphological analyses were obtained from field samples, the observed differences in size could be attributed to environmental factors such as water temperature (larger sizes in cooler waters [83]), food availability (increased body size with abundant resources [84]), salinity (higher salinities generate smaller sizes [85]), or the presence of vertebrate predators (removal of larger and/or pigmented copepods [86,87]). However, we observed that differences in size and color persisted even after culturing at common laboratory conditions for several months. When we compared the size of the Mexican populations with those reported for *L*. *sicilis* from the Great Lakes [78,82,84,88,89] the latter were larger (Table 2B), but this information is not enough to attribute such differences to environmental conditions (lower temperature and salinity) or to species differences.

However, differences in pigmentation (i.e., reddish in Atexcac and El Carmen, colorless in Quechulac and La Preciosa) observed in field and laboratory animals could be interpreted as the seasonal changes recognized within this species by Forbes [26]. These color variations could result from the foods they consumed [84]. So, we could not eliminate the possibility that phenotypic plasticity may occur in wild Mexican populations owing to temporal changes in available food resources. However, all four populations were cultured under the same illumination and feeding regimes (i.e., common garden experiment), and they produced distinctive pigmentation patterns, suggesting geographic variation among populations. Further research is needed to elucidate if this pattern is related to a trade-off between protection from ultraviolet radiation and avoidance from visual predators [90,91], which are only present in Quechulac and La Preciosa.

Genetic divergence in mtDNA COI sequences among populations differed by <0.5%, which was lower than the congeneric average distance of 8.38% [92] and 17.84% [93] found among lacustrine copepods from Mexico, or the threshold of 0.16 subst./site proposed for species delimitation within Crustacea [94]; thus these differences were not large enough to warrant separating populations from the Oriental Basin lakes into several species. Nonetheless, according to the same criterions the group is clearly separated (average distances >20%) from the other species included in the analysis. The lack of material of *L*. *sicilis* from the Great Lakes precluded morphological and genetic comparisons, so we cannot conclude if populations from Oriental Basin belong to the same species or not.

A current paradigm states that the passive dispersal of resting structures of zooplankters among closely located lakes is high enough to maintain a significant gene flow among populations [95]. However, our results show that each population has a distinct genetic structure characterized by presence of particular, unshared mtDNA COI haplotypes and absence or restriction of gene flow, with limited dispersal. Moreover, there was a remarkable pattern of allopatric fragmentation between the population from Atexcac and the other three populations, as haplotypes found in Atexcac were completely idiosyncratic. From a geological point of view, Oriental Basin lakes are relatively recent (~40,000 ybp) [38]; thus, considering that the rate of COI divergence (as a molecular clock) has been calculated at ~1.4% per My [96], it is unlikely that the genetic divergence we found among populations (0.23–0.43%) originated within these lakes. Thus, the genetic clustering may be the result of three processes leading to reduced gene flow: 1) differential colonization events associated with priority effects, 2) a build-up of local adaptation to environmental conditions in each lake by genetic variants that do not allow the entry of new genotypes, and 3) the (related) reduced fitness of migrants in habitats to which they are poorly adapted. This may have resulted in further differentiation among nearby populations as they adapted to different environments [9,97–99], as we will discuss further here below.

Similar patterns of genetic differentiation among local populations of freshwater invertebrates have been detected [97,100,101], involving weak gene flow as a consequence of low dispersal rates even at small spatial scales in cladocerans [e.g., [102]. Some others indicate the existence of microgeographic heterogeneity, the persistence of founder events and the development of microendemism in copepods [13,22], patterns that occur especially in taxa inhabiting waterbodies with different salinities. Thus, salinity is associated with increased rates of molecular evolution, promoting local genetic divergence in halophilic plankton [16,103], as it seems to be occurring in these Oriental Basin copepods.

### Ecological divergence: Specialization, local adaptation and selection against immigrants

Results from transplant experiments showed a consistent pattern of adaptive divergence among three populations inhabiting three distinct environments. Though there were significant effects of salinity and population on several variables (indicating overall plasticity to salinity or genetic clustering of populations, respectively), and the interaction term was always significant implying existence of genetic differentiation among populations in plasticity [73], i.e., each population responded differently to each salinity scenario. Populations of copepods from the deep, permanent lakes of relatively constant salinity (La Preciosa, Atexcac) are highly specialized genotypes because they performed best at their native salinities and experienced more or less dramatic decreases at foreign conditions. On the other hand, because El Carmen is a temporary playa lake that experiences wide salinity fluctuations, we expected to find a generalist genotype adapted to a wide range of conditions. Interestingly, the performance of several indicators of fitness was severely impaired at the lowest salinity; but at the other extreme (relatively high salinity; ≥6.5 g L^-1^) the negative effects on some variables were not significant. Thus, at least the genotypes collected when salinity at El Carmen was 3.8 g L^-1^ exhibited a less specialized, but not completely generalist, profile that overlaps partially with the performance of organisms from Atexcac but not from La Preciosa.

Thus, according to the ‘local vs. foreign’ criterion [74] the population inhabiting La Preciosa has developed a strong local adaptation to the lowest salinity, as evidenced by the performance of genotypes from El Carmen and Atexcac which was always inferior at that salinity. In contrast, although copepods from Atexcac apparently inhabit the best environment available for them, their performance at their local salinity was only slightly better or even surpassed by individuals from El Carmen, giving a weak pattern of local adaptation. Moreover, as La Preciosa and Atexcac genotypes are strongly adapted to their native conditions, performance of individuals from El Carmen was usually the best at the intermediate salinity, satisfying also the criterion for local adaptation in reproductive variables.

Finally, in accordance with the observed pattern of divergent adaptation, we confirmed that potential immigrants from locally specialized populations (La Preciosa, Atexcac) are poorly adapted to alternate environments [11], greatly reducing the probability of gene flow between them and with organisms migrating to El Carmen. In contrast, we found that organisms from El Carmen migrating to Atexcac would not experience significant negative selection and thus the potential of gene flow could still be considered. However, there is no evidence of such gene flow, as reflected by the differential distribution of COI haplotypes discussed before, a result that deserves further investigation. Overall, ecophysiological data show that the pattern of lack of gene flow among populations revealed by the haplotype analyses is explained at least partially by the inability of migrants to cope with alternate salinity conditions. This comprises an effective prezygotic barrier to reproduction. However, though the chances for reproduction between populations in natural conditions are scarce, the question of whether speciation has proceeded sufficiently to disrupt reproductive compatibility to any small degree among populations still remains. We consider that question below.

### Reproductive compatibility

Variation in body size among the populations was not an impediment to interpopulation mating, resulting in successful interbreeding in the intermediate salinity, even though copepods from La Preciosa and Atexcac were cultured in suboptimal conditions. Thus, divergent adaptation of populations has not produced reproductive incompatibility and according to the biological species concept [104–106] they constitute the same species and not a complex of cryptic species [13,34]. However, further analysis is needed to determine the long-term success of interpopulation offspring. To do this we would need to follow survivorship, development, and fertility of the resulting hybrids beyond the F1 generation [34,107]. This may reveal postzygotic barriers [108], such as physiological deficiencies, morphological malformations or infertility [109].

Concurrently, our results on biological fitness and reproductive compatibility are consistent with the pattern outlined by the molecular analyses. Although these populations may still be considered a single species, they have diverged somewhat as a consequence of disruptive selection (differential salinity conditions). The separation of the populations has resulted in unique local adaptations that now limit their ability to disperse to neighbor lakes.

### Speciation along the salinity gradient?

Salinity affected fitness characters throughout the life history, and more so in copepods inhabiting perennial lakes than ephemeral lakes. Three different phenotypes have evolved: two specialized phenotypes in the lakes of constant salinity, each adapted to divergent local conditions, and one intermediate generalist phenotype in a temporary lake where salinity fluctuates throughout the season. The invasion of diverse environments involves challenging physiological trials and high energetic costs to migrant copepods coming from saline to fresh water or vice versa [14]. Thus, osmoregulatory capacity should be under strong adaptive selection [110]. Individuals that are capable of surviving in salinity-fluctuating environments like El Carmen increase their probability of successful colonization into other lakes. On the other hand, if salinity is constant, the ability to osmoregulate over a wide interval of conditions is not advantageous, and the variants that perform better at the local salinity would be selected over the generations, reducing or eliminating plasticity [100]. This may account for the narrow tolerance observed in copepods from La Preciosa and Atexcac.

Passive dispersal of resting eggs of copepods among lakes by wind or waterfowl (as *Fulica americana* and some species of Anatidae that inhabit these lakes) [95] even if frequent, is not enough to allow a noticeable migration of individuals among divergent patches. For a successful colonization event, immigrants should withstand both biotic (e.g., interactions with predators and/or competitors) and abiotic conditions (e.g., different salinity, hydrodynamic regime, etc.) throughout its entire life cycle, from hatching to the production of viable offspring and the long-term population establishment. However, our results demonstrate that specialized phenotypes are poorly suited as migrants, with quite reduced probabilities of mating in alternate environments. This explains the molecular evidence for restricted gene flow among lakes over the years and the persistence of founder events, as well as the pattern of allopatric fragmentation we found [9,97,111], and supports the assertion that natural selection against immigrants is an effective reproductive barrier between populations evolving in divergent environments [112].

In summary, the four neighbor populations of copepods analyzed, although with low divergence, are genetically structured, indicating ineffective dispersal and restricted gene flow reinforced by an ecologically based divergent selection. Moreover, the prezygotic isolation among populations due to immigrant inviability is indicative of an advanced stage within the “speciation continuum” [11, 108, 113]. Preliminary results of ongoing experiments indicate that although hybrids from La Preciosa and Atexcac survive to sexual maturity, the F2 generation is no longer viable. If the current conditions persist for a longer time, ecological speciation may reach completion, generating perhaps three different biological entities, one inhabiting Atexcac lake, another distributed in Quechulac and La Preciosa and the other at El Carmen.

This study showed how ecologically based divergent selection may explain diversification patterns in lacustrine copepods. Our next step is to perform a finer-level molecular analysis using neutral markers and genomic methods [114,115]. This will allow us to relate variation in genomes to environmental adaptation [116] to better understand the processes underlying adaptive divergence in lacustrine organisms.

## Supporting Information

S1 Table Generalized linear model on the accumulated hatching of the three resting egg banks of *L*. cf. *sicilis*. Analysis was performed averaging the last five days of the experiment. Binomial distribution and a logit link function were assumed. *p* value means *p* Chi-square distribution values.
(DOCX)

S2 Table Effects of salinity, population and their interaction on reproductive variables in intrapopulation mating. Data were analyzed by means of non-parametric Scheirer–Ray–Hare tests.
(DOCX)

S3 Table Effects of the origin of male, origin of female and their interaction on reproductive compatibility in interpopulation mating trials. Egg ratio data were analyzed by mean of a two-way ANOVA, while relative hatching and hatching success by non-parametric Scheirer–Ray–Hare tests.
(DOCX)
