# Supplementary material for: From Local Adaptation to Ecological Speciation in Copepod Populations from Neighboring Lakes
Source: PLoS One. 2015 Apr 27;10(4):e0125524. doi: 10.1371/journal.pone.0125524 (PMC4411077; doi:10.1371/journal.pone.0125524)
Supplement: S1 Table — Analysis was performed averaging the last five days of the experiment. Binomial distribution and a logit link function were assumed. p value means p Chi-square distribution values. (DOCX) [file pone.0125524.s001.docx]

**Table S1.** **Generalized linear model on the accumulated hatching of the three resting egg banks of *L.* cf. *sicilis*.** Analysis was performed averaging the last five days of the experiment. Binomial distribution and a logit link function were assumed. *p* value means *p* Chi-square distribution values.

| **Source** | Df | Deviance residual | *P* |
| --- | --- | --- | --- |
| Salinity | 2 | 80.647 | <0.001 |
| Population | 2 | 78.992 | 0.437 |
| Salinity × population | 4 | 67.131 | 0.018 |
