# Supplementary material for: From Local Adaptation to Ecological Speciation in Copepod Populations from Neighboring Lakes
Source: PLoS One. 2015 Apr 27;10(4):e0125524. doi: 10.1371/journal.pone.0125524 (PMC4411077; doi:10.1371/journal.pone.0125524)
Supplement: S2 Table — Data were analyzed by means of non-parametric Scheirer–Ray–Hare tests. (DOCX) [file pone.0125524.s002.docx]

**Table S2.** **Effects of salinity, population and their interaction on reproductive variables in intrapopulation mating**. Data were analyzed by means of non-parametric Scheirer–Ray–Hare tests.

| **Source** | SS/MS_total_ | Df | *P* |
| --- | --- | --- | --- |
| **Egg ratio** |  |  |  |
| Salinity | 0.926 | 2 | 0.629 |
| Population | 17.533 | 2 | <0.001 |
| Salinity × population | 22.815 | 4 | <0.001 |
|  |  |  |  |
| **Relative hatching** |  |  |  |
| Salinity | 0.417 | 2 | 0.812 |
| Population | 13.454 | 2 | 0.001 |
| Salinity × population | 29.267 | 4 | <0.001 |
|  |  |  |  |
| **Hatching success** |  |  |  |
| Salinity | 4.175 | 2 | 0.124 |
| Population | 0.905 | 2 | 0.636 |
| Salinity × population | 33.787 | 4 | <0.001 |
