# Supplementary material for: From Local Adaptation to Ecological Speciation in Copepod Populations from Neighboring Lakes
Source: PLoS One. 2015 Apr 27;10(4):e0125524. doi: 10.1371/journal.pone.0125524 (PMC4411077; doi:10.1371/journal.pone.0125524)
Supplement: S3 Table — Egg ratio data were analyzed by mean of a two-way ANOVA, while relative hatching and hatching success by non-parametric Scheirer–Ray–Hare tests. (DOCX) [file pone.0125524.s003.docx]

**Table S3.** **Effects of the origin of male, origin of female and their interaction on reproductive compatibility in interpopulation mating trials.** Egg ratio data were analyzed by mean of a two-way ANOVA, while relative hatching and hatching success by non-parametric Scheirer–Ray–Hare tests.

| **Source** | Df | F | *P* |
| --- | --- | --- | --- |
| **Egg ratio** |  |  |  |
| Male | 2 | 0.149 | ..0.862 |
| Female | 2 | 13.611 | <0.001 |
| Male × female | 3 | 0.880 | ..0.455 |
|  |  |  |  |
| **Source** | SS/MS_total_ | Df | *P* |
| **Relative hatching** |  |  |  |
| Male | 1.184 | 2 | 0.553 |
| Female | 25.053 | 2 | <0.001 |
| Male × female | 5.191 | 3 | 0.158 |
|  |  |  |  |
| **Hatching success** |  |  |  |
| Male | 0.482 | 2 | 0.790 |
| Female | 4.213 | 2 | 0.1217 |
| Male × female | 6.015 | 3 | 0.077 |
